# 

**Published:** 1882-11

**Authors:** 


### CONTENTS.

### COMMUNICATIONS.

The Centralization of Energy .................................................. 523
The Etiology and Pathology of Dental Diseases: What We Know, and What We Would Like to Know .................................. 546
Success in the Profession of Dentistry.......................................... 554
Teeth .................................................................. 561

### SELECTIONS.

Crural Neuralgia among Dentists.................................................. 565
Counter-Irritation .................................................................. 566
The Topical Uses of Tannic Acid ................................................ 567
Peroxide of Hydrogen in Surgery................................................. 568
Sir Eramus Wilson's Munificent Gift to Margate ........................... 569

### EDITORIAL.

Continuous Gum Dentures.......................................................... 569
Ohio State Dental Society ........................................................ 570
Transactions—Michigan State Dental Society.................................. 572
Transactions—Ohio State Dental Society ...................................... 572
Examinations .......................................................................... 572

